# Distinguishing Inner and Outer-Sphere Hot Electron Transfer in Au/p-GaN Photocathodes

**DOI:** 10.1021/acs.nanolett.4c04319

**Published:** 2024-11-01

**Authors:** Fatemeh Kiani, Alan R. Bowman, Milad Sabzehparvar, Ravishankar Sundararaman, Giulia Tagliabue

**Affiliations:** † Laboratory of Nanoscience for Energy Technologies (LNET), STI, École Polytechnique Fédérale de Lausanne, 1015 Lausanne, Switzerland; ‡ Department of Materials Science & Engineering, Rensselaer Polytechnic Institute, 110 Eighth Street, Troy, New York 12180, United States

**Keywords:** plasmonic photocatalysis, Au/GaN photocathode, hot electron transfer, inner-sphere, outer-sphere, scanning electrochemical microscopy

## Abstract

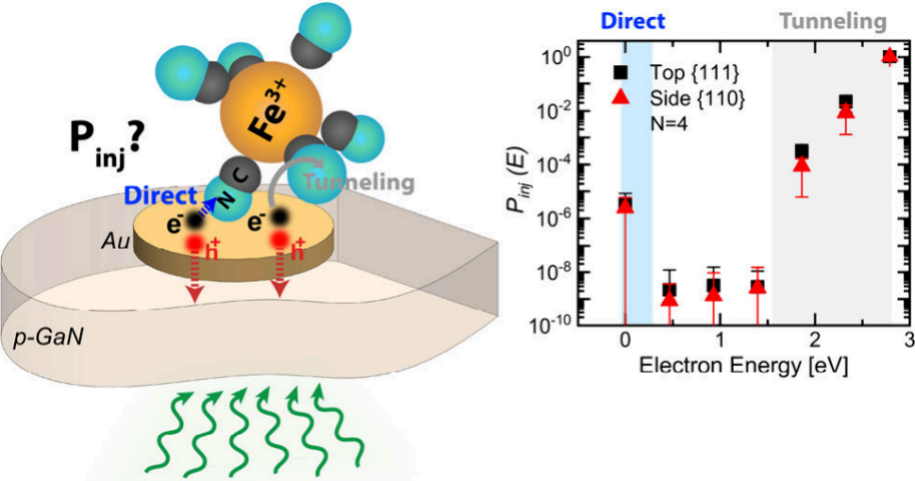

Exploring nonequilibrium hot carriers from plasmonic metal nanostructures is a dynamic field in optoelectronics, with applications including photochemical reactions for solar fuel generation. The hot carrier injection mechanism and the reaction rate are highly impacted by the metal/molecule interaction. However, determining the primary type of reaction and thus the injection mechanism of hot carriers has remained elusive. In this work, we reveal an electron injection mechanism deviating from a purely outer-sphere process for the reduction of ferricyanide redox molecule in a gold/p-type gallium nitride (Au/p-GaN) photocathode system. Combining our experimental approach with ab initio simulations, we discovered that an efficient inner-sphere transfer of low-energy electrons leads to an enhancement in the photocathode device performance in the interband regime. These findings provide important mechanistic insights, showing our methodology as a powerful tool for analyzing and engineering hot-carrier-driven processes in plasmonic photocatalytic systems and optoelectronic devices.

Plasmonic catalysis shows great promise in improving reaction rates and selectivity across various catalytic applications.^1−5^ Three potential mechanisms have been shown to drive reactions: enhanced electromagnetic near fields, temperature increase at the metal/liquid interface, and transfer of excited hot carriers.^6−9^ The last of these processes has attracted attention for the possibility of steering chemical reactions by accessing highly excited unoccupied states.^5,10−12^ Practical applications of hot carrier devices requires a full understanding of plasmonic hot-carrier-driven processes including plasmon excitation, hot carrier generation, and injection at interfaces. However, physical understanding of hot carrier transport and injection mechanisms at metal/molecule and metal/semiconductor interfaces in these devices remained elusive.

Hot carrier collection schemes typically involve the formation of an interfacial Schottky barrier between plasmonic metals (e.g., Au) and wide band gap semiconductors (e.g., TiO_2_).^8^ Indeed, depositing gold nanostructures on a wide bandgap semiconductor allows the extraction of one hot charge carrier to the semiconductor and the use of the other one to drive an oxidation or reduction reaction at a metal/liquid interface. It has been recently shown that the energy of distribution of the hot carriers and their ballistic transport to the metal/semiconductor or metal/molecule interface play a critical role for the efficiency of plasmonic hot carrier devices.^8,13,14^ In addition to the energy distribution of hot carriers, the interaction of molecules with the metal surface and the injection mechanism of the hot carriers to the molecule will play a role. The two main reaction pathways are outer- and inner-sphere reactions. In an outer-sphere reaction, the hot carrier transfer between the metal and the molecule occurs at a plane separated by a solvent layer from the metal surface (Figure 1.a).^15^ This introduces a tunneling barrier for carrier transfer to the molecule.^16^ Conversely, in an inner-sphere reaction carrier transfer can take place through a bridging ligand of the adsorbed molecule bonds to the metal surface.^15,17^ Thus, energy-dependent hot carrier injection processes and rates of the reaction are expected to be highly impacted by the metal/molecule interaction.^5,11,18−20^

**Figure 1:**
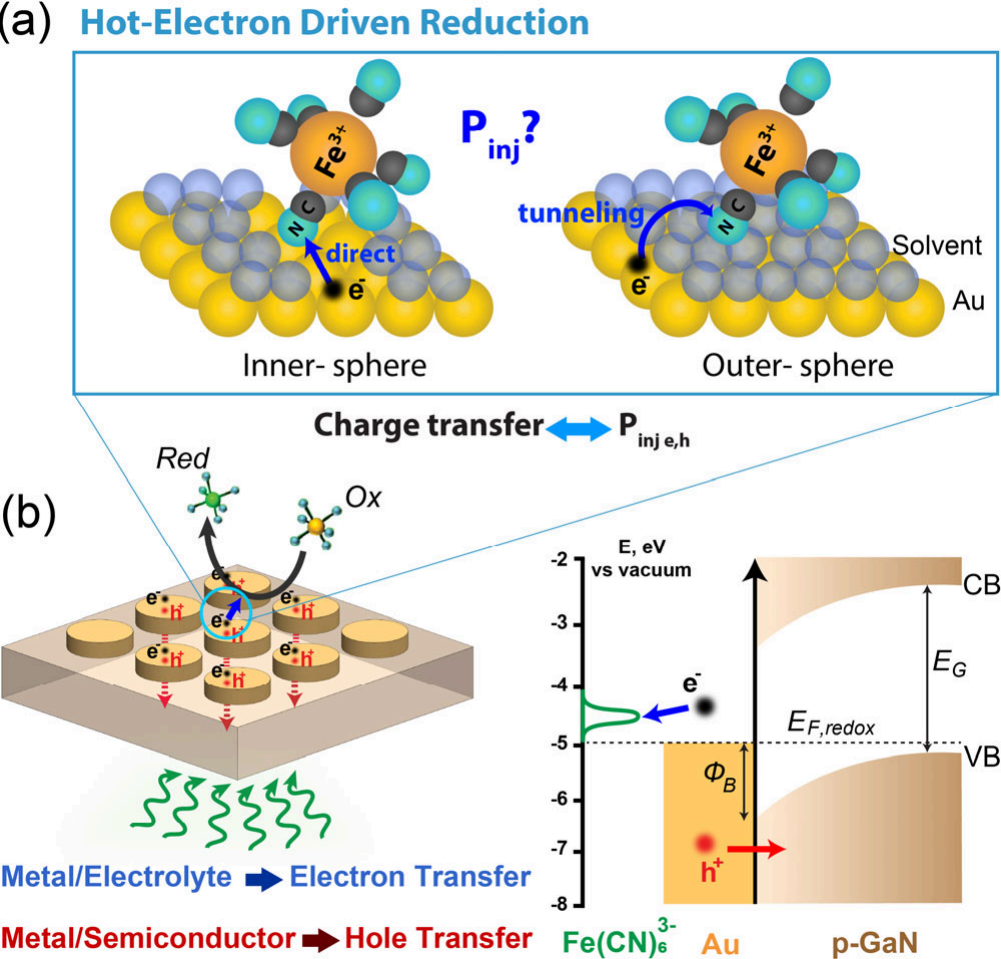
Schematic of interfacial hot carrier collection in plasmonic metal/semiconductor heterostructure devices. (a) Injection mechanisms of hot electrons across the metal/molecule interface. (b) Au NDs/p-GaN photocathode in contact with an oxidant (*Ox*) molecule, together with the band alignment showing hot-hole and hot-electron collection across the Au/p-GaN and Fe(CN)_6_^3–^ interfaces.

In this work, we study plasmonic Au/p-GaN photocathodes to drive reduction of ferricyanide redox molecule, Fe(CN)_6_^3–^, while using a p-type semiconductor to collect the hot holes to reduce charge recombination. Leveraging our unique methodology,^8^ which combines scanning electrochemical microscopy (SECM) and ultrathin monocrystalline gold nanodisk antennas, we determine the energy-resolved injection probability of hot electrons and reveal two coexisting mechanisms of charge transfer at the metal/molecule interface: an outer-sphere transfer of high-energy electrons that inject into the Fe(CN)_6_^3–^ molecule through a tunneling process, and an inner-sphere transfer of low-energy electrons that directly inject into the LUMO of the molecule (Figure 1.a). We suggest the latter comes from the higher affinity of the Fe(CN)_6_^3–^ molecules to adsorb on the surface and we show how it is also impacted by the used electrolyte. This comprehensive mechanistic understanding highlights the importance of leveraging careful optical and photoelectrochemical methods to unravel complex charge transport processes at interfaces in plasmonic hot-carrier-driven photodetection and photocatalytic systems, with major implications for plasmonic artificial photosynthesis devices.^3,4^

We fabricated plasmonic photocathodes consisting of an array of single-crystalline Au nanodisks (Au NDs) with diameters of the order of tens of nanometers on an optically transparent p-type GaN (p-GaN) wide bandgap semiconductor (bandgap ≃3.4 eV,^21^ see Supporting Information 1) (Figure 1.b). The Au NDs were obtained by patterning individual Au microflakes^22^ with thicknesses ranging from 14 to 27 nm. Thus, their top surface consistently exhibits {111} orientation. The Au NDs are in contact with an electrolyte containing a reversible redox molecule, Fe(CN)_6_^3–^ (ferricyanide, the oxidized form, *Ox*) which, based on the energy diagram of the system, enables hot-electron collection during photochemical reduction (Figure 1.b). This reduction is expected to proceed via a one-electron transfer, nonpurely outer-sphere mechanism with fast kinetics.^17,23−25^ The chosen molecule also does not absorb visible light (Figure S2, 470–832 nm) hence only charge carriers generated by light absorption in the Au NDs can contribute to the photochemical processes.

Scanning photoelectrochemical microscopy (photo-SECM)^8^ is an exquisitely sensitive technique that has emerged as a promising method to quantify the photochemical response of plasmonic photocatalysts.^23,26^ As shown in Figure 2.a, we performed photo-SECM measurements in substrate generation/tip collection (SG/TC) mode on Au ND arrays with disk thicknesses of 14, 16, and 18 nm, and average diameters of 52, 68, and 67 nm, respectively. All tested samples were kept at open circuit condition and illuminated from the bottom with a collimated laser beam (∼30 μm diameter). The ferricyanide photoreduction occurred only within the illuminated area of the samples due to hot-carrier generation and hot-electron transfer at the Au/electrolyte interface. The evolved reductant species were detected (i.e., oxidized) at the Pt ultramicroelectrode (UME) tip, which was kept at an oxidation potential of 0.4 V vs Ag/AgCl and positioned close to the sample surface. Following a previously established methodology^8,23^ (Supporting Information S3), we measured the UME tip oxidative current at each excitation wavelength λ as a function of the laser power to determine the substrate photocurrent (i_sub,photo_(λ)). Control experiments on a bare p-GaN substrate in the absence of the Au NDs showed an extremely low photoresponse at short wavelengths (470 nm-480 nm) and high excitation intensities (Figure S5), with no response at longer wavelengths. This contribution was subtracted from the Au/p-GaN photocurrent. Dividing i_sub,photo_(λ) by the incident laser power (P_in_(λ)), we obtain the external quantum efficiency (EQE) spectrum of each plasmonic photoelectrode (Figure 2.b, also called incident photon to current collection efficiency or IPCE). Interestingly, all EQE curves exhibit a very steep increase for photon energies higher than 2.4 eV as well as a small peak in the range 1.6–1.8 eV, associated with the characteristic plasmon resonance of each structure. This is confirmed from the absorption spectra of our devices, which we determine using a combination of microscale absorption measurements in air and electromagnetic simulations^27^ (Supporting Information 2).

**Figure 2:**
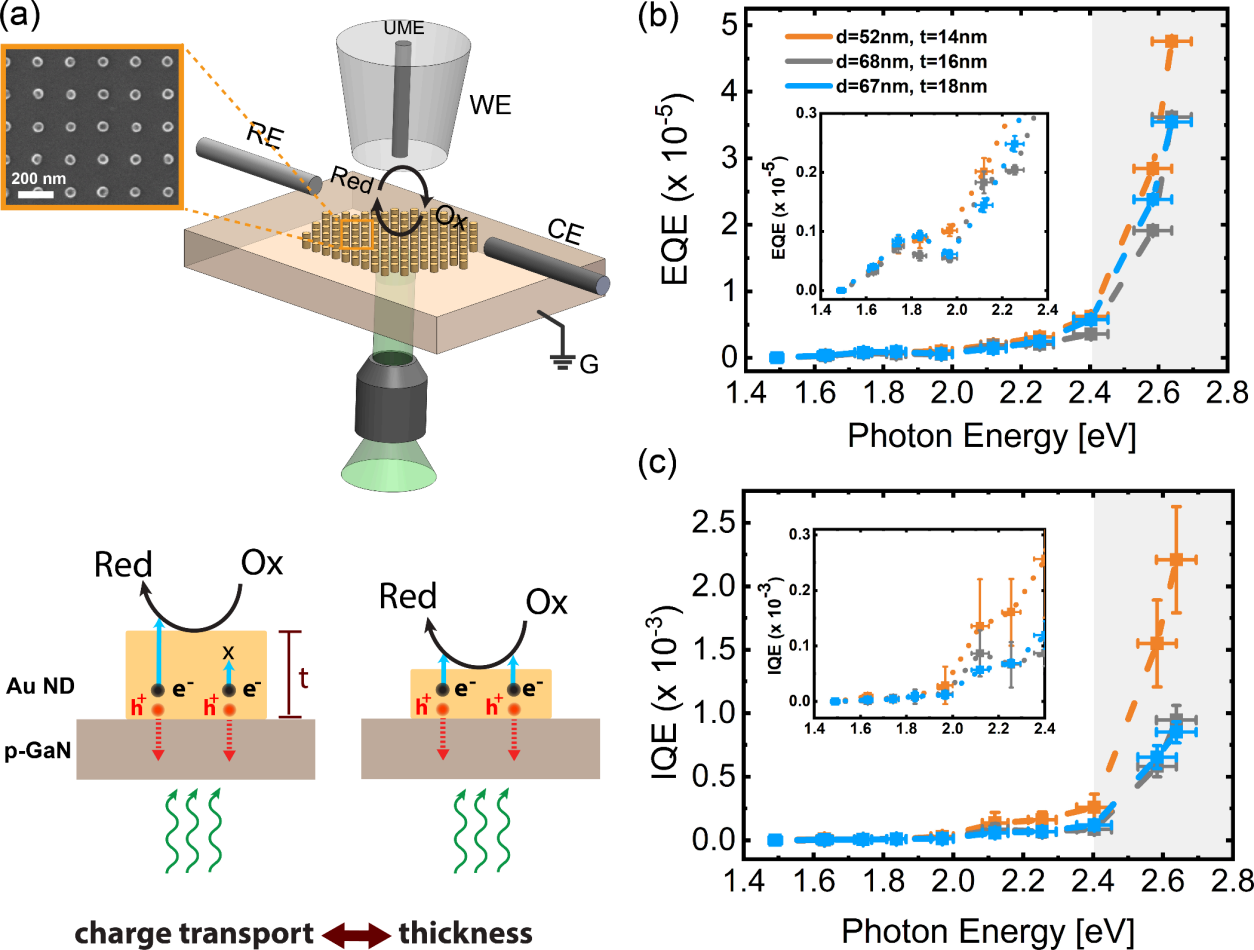
Liquid-state photochemical measurement results. (a) Schematic of the designed plasmonic heterostructure and photo-SECM configuration in a substrate generation/tip collection (SG/TC) experiment mode. A fabricated Au NDs array from a single-crystalline Au microflake on p-GaN substrate is in contact with an electrolyte contacting 4 mM Fe(CN)_6_^3–^ (oxidant, *Ox*) and 0.25 M KCl. A 1.45 μm-radius Pt UME tip is positioned 3 μm away from the substrate. The UME tip is biased at 0.4 V vs Ag/AgCl (reference electrode, *RE*), and the substrate is at open circuit and grounded. Light is incident on the plasmonic Au NDs array from the bottom. The reverse reaction happens at the tip electrode and substrate surface. The current is measured through the tip working electrode (*WE*). A Pt wire is used as a counter electrode (*CE*) to complete the circuit. The inset shows the SEM image of the Au ND array with an average diameter of 52 nm and thickness of 14 nm. The side-view schematics in (a) illustrate the direction of carrier transfer at interfaces and represent the thickness effect on charge transport. Experimentally determined (b) external quantum efficiency (EQE) and (c) internal quantum efficiency (IQE) spectra for the fabricated heterostructures having different Au ND dimensions. The inset in (b) and (c) shows the magnified view of the EQEs and IQEs from 1.4 to 2.4 eV, respectively. The gray shaded areas depict the purely interband region,^28,29^ and the dashed lines are a guide to the eye in panels (b) and (c).

Finally, the internal quantum efficiency of the devices is calculated dividing the EQE spectra by the absorption ones (Figure S1). Interestingly, the IQE spectra are largely featureless from 1.4 to 2 eV (no plasmon resonance).^8,13^ They instead show a minor bump at 2.1 eV and reach their maximum efficiency in the interband regime, with the best performance for the thinnest (14 nm thick) NDs device (Figure 2.c). This is a surprising result because for an outer-sphere reaction the electron tunneling probability increases with the carrier energy and in the interband regime hot electrons in Au preferentially have low energies.^13^ Thus, a decrease in the number of collected hot electrons per absorbed photon would be expected in the energy range above 2.4 eV.

To understand our photochemical results, which involve simultaneous collection of electrons and holes, the role of hot hole removal at the metal/semiconductor interface must be first clarified. We thus perform solid-state photocurrent measurements using a separate plasmonic hot hole photodiode device consisting of an array of monocrystalline Au stripes (15 and 27 nm thickness) on the same p-GaN substrate (Figure 3.a, Supporting Information S4). Similarly to the photochemical device, we measured absorption as well as the photocurrent as a function of the excitation wavelength, overall determining the external and internal quantum efficiencies of these devices (Figure 3.b). We observe that the steeply increasing IQE values of the photodiode devices at high photon energies is consistent with theoretical predictions of the energy distributions of photoexcited hot carriers generated via interband transitions.^29^ In fact, as previously discussed, as the incident photon energy is continually increased above the onset of the interband threshold for Au (*h*ν ≈ >1.8 eV), an ever-increasing fraction of hot holes are generated within the d-bands of the metal. These hot holes possess enough energy to overcome the interfacial Schottky barrier (Φ_B_ = 1.3 eV, Figure S6.b) and effectively inject into the p-GaN valence band. Overall, our photodiode results are in excellent agreement with prior reports.^14^

**Figure 3:**
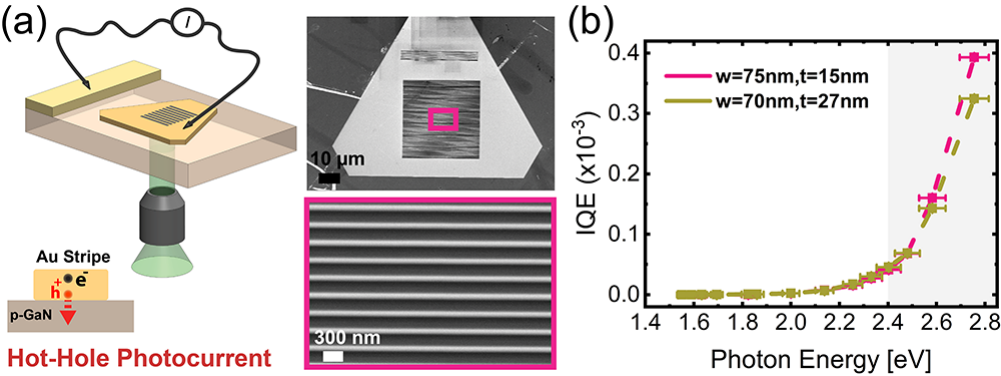
Solid-state photocurrent measurement results. (a) The inset shows the schematic of the designed plasmonic hot hole photodiode heterostructure and solid-state measurement configuration. A stripe Au pattern is fabricated from an SC Au MF on p-GaN substrate together with a 100 nm thick sputtered Au film Ohmic contact. Light is incident on the plasmonic Au stripe array from the bottom and the photocurrent is collected by two microcontact probes electrically connected to the Au flake and the sputtered Au contact pad. The side-view schematic illustrates the direction of hole transfer at the Au/p-GaN interface. SEM image on the right shows a 30 × 30 μm^2^ stripe array from a 15 nm-thick Au MF together with the higher magnification SEM image of the fabricated Au stripe array. The average stripe width and thickness are 75 and 15 nm, respectively. The array periodicity is 230 nm. (b) Measured IQE spectra of the fabricated heterostructures having thicknesses of 15 and 27 nm. The gray shaded areas depict the purely interband region^28,29^ and the dashed lines are a guide to the eye.

When we compare the IQE spectra of the photocathode and photodiode devices (Figures 2c and 3b), an interesting observation arises: they both exhibit a remarkably similar trend, and the magnitude of the IQE for the photocathodes is surprisingly higher than the IQE of the hot-hole collection in photodiodes. We performed a photo-SECM experiment at a concentration of Fe(CN)_6_^3–^ that is four times lower (i.e., 1 mM oxidant). Figure 4.a shows the IQE of the 14 nm Au NDs/p-GaN photocathode in 1 mM and 4 mM Fe(CN)_6_^3–^ electrolyte, with the brown curve scaled by a factor 4. We clearly observe a change in the IQE magnitude directly proportional to the concentration of oxidant, while preserving the photon-energy dependence (the same IQE trend). Thus, the IQE of the photocathodes is not limited by hot hole collection at the solid–solid interface. The higher magnitude of the IQE for the photocathode is instead likely due to a fast and effective electron collection at the metal/molecule interface.

**Figure 4:**
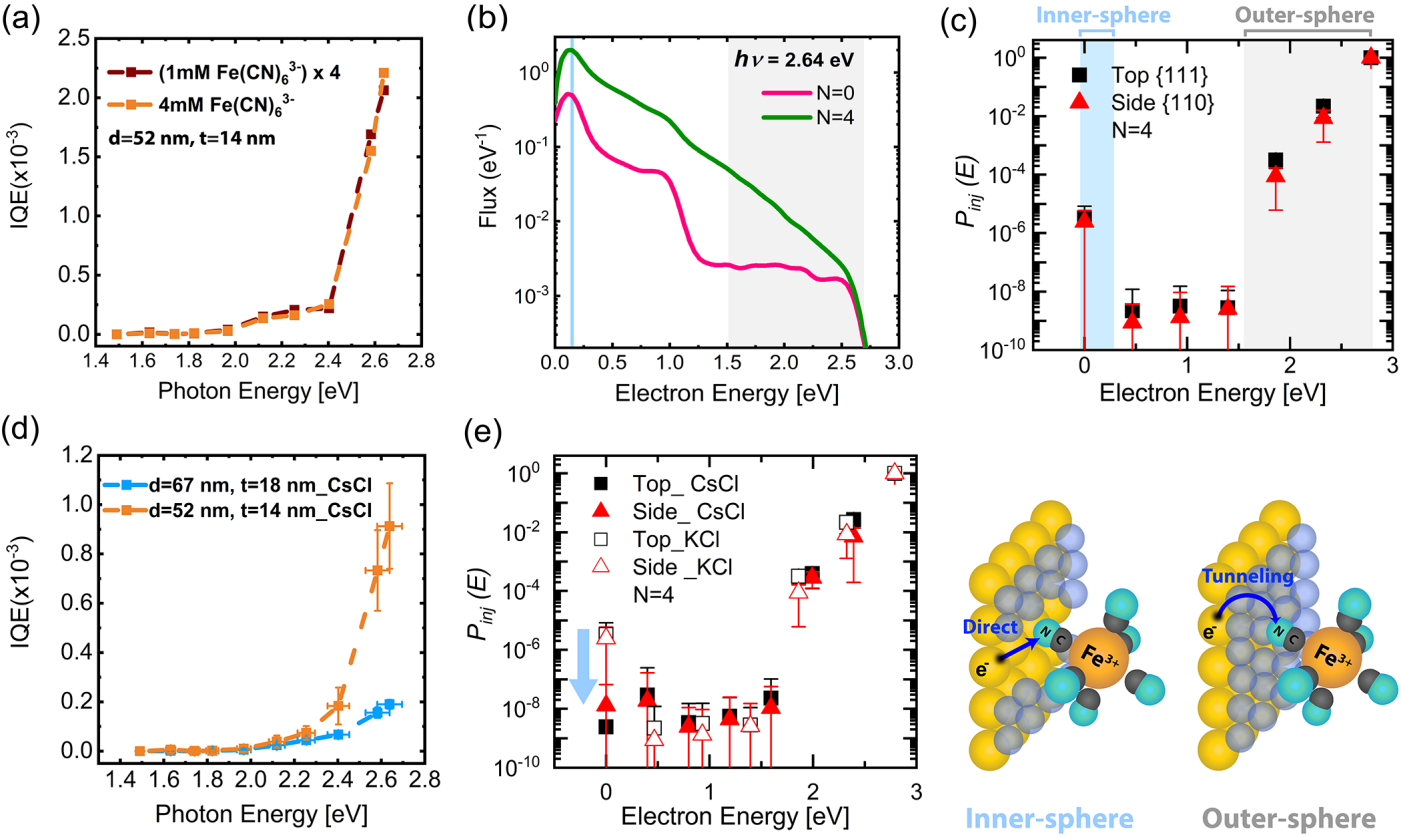
Hot electron generation, transport, and injection in Au ND/p-GaN photocathode devices. (a) IQE of the 14 nm Au NDs/p-GaN photocathode in 1 mM (x4) and 4 mM Fe(CN)_6_^3–^/0.25 M KCl electrolyte concentrations. The IQE magnitude is directly proportional to the concentration of Fe(CN)_6_^3–^, while the energy dependence remained conserved. (b) Calculated energy resolved electron fluxes reaching the top surface of 14 nm thick Au NDs directly (*N* = 0) or upon scattering (*N* = 4) under illumination at 470 nm (2.64 eV). The blue line shows the position of the LUMO with respect to the Fermi level (0.19 eV). The gray shaded area shows the tiny flux of high-energy hot electrons (1.5–2.64 eV). (c) Injection probability (*P*_*inj*_*(E)*) for hot electrons collected from the top {111} and side {110} facets of the Au NDs after 4 scattering events. The *P*_*inj*_*(E)* plots are extracted from the fitting approach using the energy-resolved hot electron fluxes and experimentally determined IQEs of Au NDs heterostructures. The direct (inner-sphere) and tunneling (outer-sphere) charge injection mechanism is indicated for the low-energy and high-energy regions, respectively. (d) IQE spectra of 14 and 18 nm Au NDs/p-GaN photocathodes in 4 mM Fe(CN)_6_^3–^/0.25 M CsCl electrolyte. (e) Calculated injection probabilities for the measurements performed in the CsCl electrolyte (filled symbols) together with the measurements in the KCl electrolyte (empty symbols). The dashed lines are a guide to the eye in panels (a) and (d).

To understand how the electron collection controls the IQE in this system and explain the unexpected trend of IQE with photon energy (Figure 2.c), we employed the Non-Equilibrium Scattering in Energy and Space (NESSE) transport model.^8,30^ Validated by several prior works,^14,30,31^ NESSE efficiently calculates the energy-resolved fluxes of hot carriers that reach the surfaces of a plasmonic nanoantenna and it includes all possible scattering mechanisms, including Auger scattering.^32^ In this case we determine the energy-resolved flux of hot electrons that reach the metal/electrolyte interface after *N* scattering events as a function of the photon energy. Figure 4.b shows the calculated energy-resolved cumulative electron fluxes reaching the top surface of 14 nm thick Au NDs directly or upon scattering under illumination at 470 nm (2.64 eV, carriers are generated via interband electron transitions). For each photon energy (*ℏω*), a distribution of hot carriers exist at energies (*E*) from the Fermi level up to the photon excitation energy.^29^ The unscattered distribution (*N* = 0) shows the maximum flux for low-energy hot electrons close to the Fermi level. This portion increases after each scattering event (*N* = 1–4). On the other hand, the small flux of high-energy hot electrons (>2.4 eV) does not increase significantly with the number of scattering events.

In order to drive a photochemical reaction, the hot electrons that reach the metal/semiconductor interface must transfer to the ferricyanide molecule. The measured IQE at each photon energy is thus determined by the product of the injection probability (*P*_*inj*_*(E)*) and the electron flux (*F*_*N*_*(E, ℏω)*), integrated over all available carrier energies. Importantly, the injection probability function must be the same for all the studied Au NDs, regardless of the thickness and diameter, as the crystallinity and exposed facets remain the same. Thus, as we showed previously, a stochastic fitting of the experimental IQE spectra can estimate the injection probability of the hot electrons to the molecule (see Supporting Information 5). Figure 4.c shows the resulting *P*_*inj*_ (*E*) of hot electrons collected from the top {111} and side {110} surfaces^8,22^ of the NDs. No difference was obtained in *P*_*inj*_ (*E*) for high-energy hot electrons (>2 eV) in nonequilibrium (Figure S9, *N* = 0) and steady-state (Figure 4.c, *N* = 4) conditions. This shows the predominance of ballistic collection at high electron energies.

Interestingly, regardless of the number of scattering events considered, the energy-resolved hot electron injection probability (Figure 4.c, *N* = 4) shows the coexistence of two distinct hot electron transfer processes: (i) a tunneling contribution that exponentially increases with increasing electron energy: this is consistent with an outer-sphere electron transfer and is similar to the *P*_*inj*_ (*E*) obtained for outer-sphere hot-hole-driven oxidation of Fe(CN)_6_^4–^,^8^ and (ii) an efficient transfer of low energy electrons around the lowest unoccupied molecular orbital (LUMO) of Fe(CN)_6_^3–^ molecule, which is about 0.19 eV with respect to the Fermi level of the system (Figure 1.b). The latter contribution can be explained considering the low reduction potential or LUMO level of Fe(CN)_6_^3–^ and a different electron transfer mechanisms. Notably, surface-enhanced Raman spectroscopy (SERS) studies^17,24,25,33^ have demonstrated that electron transfer in the ferri-/ferrocyanide system cannot strictly be considered as a purely outer-sphere process and have indicated the presence of bridging CN ligands in Fe(CN)_6_^3–^ molecules adsorbed on the Au surface.^17,24^ In particular, because of the interaction between the Fe d-orbitals and the π*-antibonding orbitals of the cyanide (CN) ligands, the molecular LUMO corresponds mainly to the π*-antibonding orbital of the CN ligands.^34^ These ligands form bonds between the Au surface and at least one CN group through the lone-pair electrons on the N atoms. Thus, these interactions mainly occur in a CN antibonding orbital, enabling efficient inner-sphere electron transfer from Au to the LUMO of Fe(CN)_6_^3–^ molecule. On the other hand, it has been shown that Fe(CN)_6_^4–^ is not strongly adsorbed on the Au surface, as Fe–C≡N bonding is stronger than the C≡N bond,^24,33^ preventing the existence of such a direct charge transfer upon hot hole oxidation.^8^ Overall, the efficient inner-sphere transfer of low-energy electrons to the LUMO (≈ 0.19 eV) results in the large value of *P*_*inj*_ (*E* ≈ 0) (Figure 4.c). Combined with the large flux of low-energy hot electrons (Figure 4.b, blue line), significantly higher compared to the high-energy ones (Figure 4.b, gray area), this results in the rapid IQE growth in the interband regime (Figure 2.c, gray area).

Figure 4.c also shows a comparable *P*_*inj*_*(E)* for the hot electrons collected from the top {111} and side {110} facets of the Au NDs. This can be related to the comparatively long mean free path of hot electrons (∼10 nm for a 2 eV hot electron),^29^ which allows them to be collected more efficiently from both surfaces compared to the hot holes (see our previous work).^8^ Moreover, all the ND structures studied here are ultrathin (with thicknesses <20 nm), resulting in a uniform generation profile of hot carriers across the volume of the NDs.^8^ Our transport model and stochastic fitting approach of the injection probability reproduced our experimental data well, and almost the same computed IQE trend was obtained up to 2.5 eV (Figure S10). The larger discrepancy for the high carrier energy values is likely due to calculation errors in the very small flux of high-energy electrons obtained within this range of incident photon energy.

Importantly, it has been shown that the electrolyte used plays a key role in the adsorption of molecules on surfaces and, consequently, in the charge transfer process.^35−37^ Specifically, it was found that for ferricyanide reduction, the outer sphere character of the reaction improves when KCl supporting electrolyte is replaced with CsCl.^36^ This can be explained by the larger hydration shell, lower surface affinity and thicker electric double-layers of larger-size Cs^+^ ions as compared to K^+^ ions, preventing ferricyanide molecules to approach Au surface for an efficient cyanide ligand interaction.^36,38,39^ Therefore, we repeated our measurements on the same structures using a CsCl supporting electrolyte. Figure 4.d shows the IQEs of the 14 and 18 nm thick NDs when tested in a CsCl electrolyte. Compared to the measurements in a KCl electrolyte, the IQE curves increase more smoothly and do not present a clear step and plateau starting around 2 eV. Additionally, the overall magnitude of the IQE is lower than in the case of KCl, with a pronounced detrimental effect observed with increasing Au ND thickness. These observations suggest a change in the direct charge transfer process. In fact, the large flux of low-energy hot electrons generated by interband transitions (>2 eV photon energy) can transfer efficiently only if a direct path is available around the LUMO level (tunneling has a very low probability at low energies). Furthermore, the high-energy electrons involved in tunneling have a short mean free path, and their flux decreases significantly with increasing thickness of the NDs. Figure 4.e shows the calculated injection probabilities for the measurements performed in the CsCl electrolyte (filled symbols). In agreement with the discussion above, we observe a significantly lower probability of injection for the low-energy electrons. This confirms that the charge transfer mechanism is sensitive to the electrolyte and that the presence of Cs ions suppresses direct charge transfer, making the hot carrier-driven transformation predominantly based on the tunneling of hot electrons. Overall, these results demonstrate that our approach is capable of distinguishing nanoscopic processes in hot carrier-driven devices. They also suggest that the choice of solvent must be considered when comparing similar experimental results. Additionally, this is a factor that can be engineered to improve the efficiency of hot carrier devices.

In summary, we investigated the transport and collection of hot carriers in ultrathin (14–18 nm) single-crystalline plasmonic Au nanoantenna arrays. We studied Schottky photodiode and photocathode devices that collect hot holes (Au/p-GaN) and both hot holes and electrons (Fe(CN)_6_^3–^/Au/p-GaN), respectively. Our internal quantum efficiency (IQE) analysis showed that photocathode performance is primarily driven by hot-electron collection at the metal/molecule interface. We identified two charge transfer contributions: low-energy electrons transferring directly via an inner-sphere process and higher-energy electrons transferring through outer-sphere tunneling. The efficient collection of low-energy electrons led to a continuous increase in IQE in the interband regime. Our measurements in different supporting electrolytes revealed that charge transfer mechanism is sensitive to the electrolyte and the inner-sphere process is suppressed in the presence of the Cs ions. Additionally, we confirmed the ballistic collection of high-energy d-band holes at the Au/p-GaN interface. Our results shed light on the important mechanisms governing the transport and injection of hot carriers across interfaces in hot-carrier-driven photocatalytic systems, demonstrating the importance of our IQE analysis as powerful tool for designing efficient hot-carrier-driven devices, particularly plasmon-driven artificial photosynthetic systems. Future experiments using surface-enhanced Raman spectroscopy (SERS) will expand our understanding by identifying intermediate chemical species and reaction pathways, especially for complex reactions involving inner-sphere charge transfer.

## Data Availability

The data underlying this manuscript are available at https://doi.org/10.5281/zenodo.14003179.

## Supporting Information Available

Material synthesis and device fabrication; optical characterization of nanoantennas (absorption) and numerical modeling; photochemical characterization, EQE and IQE calculations; Au/p-GaN photodiode properties and characterization; hot carrier transport and injection, detailed IQE analysis (PDF)
