# Supplementary material for: Distinguishing Inner and Outer-Sphere Hot Electron Transfer in Au/p-GaN Photocathodes
Source: Nano Lett. 2024 Nov 1;24(50):16008–14. doi: 10.1021/acs.nanolett.4c04319 (PMC11660233; doi:10.1021/acs.nanolett.4c04319)
Supplement: Supplementary file 1 — nl4c04319_si_001.pdf [file nl4c04319_si_001.pdf]

Supporting Information

Distinguishing Inner and Outer-Sphere Hot Electron Transfer in Au/p-GaN Photocathodes

*Fatemeh Kiani[1], Alan R. Bowman[1], Milad Sabzehparvar[1], Ravishankar Sundararaman[2], Giulia Tagliabue[1]**

[1] Laboratory of Nanoscience for Energy Technologies (LNET), STI, École Polytechnique Fédérale de Lausanne, 1015 Lausanne, Switzerland

[2] Department of Materials Science & Engineering, Rensselaer Polytechnic Institute, 110 8th Street, Troy, New York 12180, USA

**E-mail: giulia.tagliabue@epfl.ch*

16 pages, 13 figures

Contents

- **Supplementary Information 1 – Material synthesis and nanofabrication**
- **Supplementary Information 2 – Optical Characterization of Nanoantennas (Absorption) and Numerical Modelling**
- **Supplementary Information 3 – Photochemical Characterization, EQE and IQE Calculations**
  - **SECM measurements**
  - **EQE and IQE determination, including error analysis**
- **Supplementary Information 4 – Au/p-GaN Photodiode Properties and Characterization**
- **Supplementary Information 5 – Hot carrier transport and injection, Detailed IQE analysis**

Supplementary Information 1 – Material synthesis and nanofabrication

The monocrystalline gold flake synthesis follows the methodology described in Ref.[1] while the nanofabrication process is a combination of those reported.[2,3] The exact details of the materials and processes used in this work are reported below for clarity and reproducibility.

GaN films grown on double-side polished sapphire (430 µm, c-plane) were provided by the Advanced Semiconductors for Photonics and Electronics (LASPE) laboratory in EPFL (1.2 µm thick Mg-doped GaN layer, ND=1x10^{19} cm^{-3} on 2.7 µm GaN non-doped buffer layer). A PMMA wet-transferring method[4] was used for transferring the single-crystalline Au microflakes (SC Au MFs)[1] onto the p-GaN substrates.

Importantly, before the transferring step, the samples were dipped in a 1:15 NH_4OH:DI H_2O solution for 30 s to remove any surface oxide layer and finally rinsed in water (30 s) and blown dry with nitrogen.[5] Briefly, fabrication process for the Au NDs/p-GaN photocathode device involved using electron-beam lithography (EBPG5000ES system) to pattern a nanodisk array on Au MFs (100 pA beam current, 480 μC/cm^2 exposure), followed by PMMA development (immersion in MIBK and IPA solutions for 1 min each). Ion beam etching (Veeco Nexus IBE350) was then used to etch the Au flake area around the nanodisks (ultra low IBE process, -10° angle). Remaining PMMA on the nanodisks was removed by immersion in pure acetone (30 min) and isopropanol (3 min), followed by rinsing with DI water and drying with N_2. For the fabrication of the Au stripes/p-GaN Schottky photodiode device, focused ion beam (FIB) milling (30 kV Ga^+ beam) was used to make a 30x30 μm^2 Au stripe array from a transferred SC Au MF onto a p-GaN/sapphire substrate. FIB milling was employed using a FIB/SEM dual-beam instrument (Zeiss CrossBeam 540). Finally, a narrow film of Ti/Au (5 nm/100 nm) Ohmic contact was deposited on the substrate surface close to the fabricated Au stripe array structure using a sputtering system (Alliance-Concept DP 650).

Supplementary Information 2 – Optical Characterization of Nanoantennas (Absorption) and Numerical Modelling

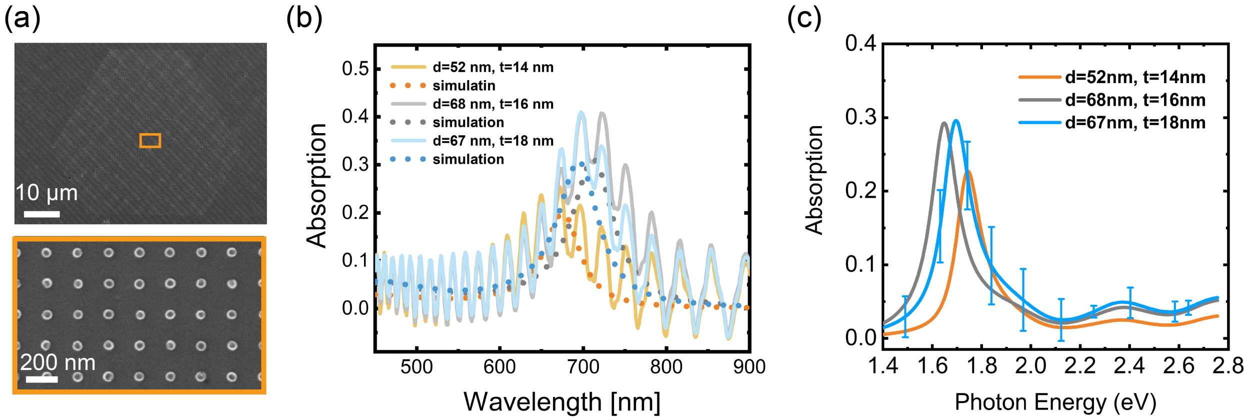

Figure S1. (a) SEM image of an entirely patterned 14 nm thick Au MF together with the higher magnification SEM image of the fabricated Au NDs array. The average ND diameter and thickness are 52 and 14 nm, respectively. The array periodicity is 200 nm. (b) The measured and simulated absorption spectra of the fabricated heterostructures having different Au ND thicknesses of 14, 16, and 18 nm in an air medium. (c) Simulated absorption spectra of the fabricated heterostructures in a water medium.

SEM image in **Figure S1.a** shows one of our fabricated ND arrays from a 14 nm thick Au MF with a lateral size of 50 µm on a p-GaN substrate together with the magnified view of the fabricated ND structure.

Absorption Measurements

We quantified experimentally the absorption spectra of the structures in air using microscale absorption measurements (**Figure S1.b**, solid lines). The methodology is explained in depth in Ref.[6]. Briefly, optical measurements were performed using an inverted microscope (Nikon Eclipse Ti2) combined with a grating spectrometer (Princeton Instruments Spectra Pro HRS-500). A fiber-coupled broadband laser-driven white light source (Energetiq LDLS™) was used to illuminate the sample from the bottom with collimated light, focused on the back focal plane (BFP) of a long working distance, high-NA objective (Nikon 60x, NA=0.7). Reflected light was directed to the spectrometer, and reflectance measurements were normalized to the reflectance of a silver mirror (ThorLabs, PF10-03-P01) and background-subtracted. Transmittance measurements were conducted with top illumination through a bright field condenser lens. The sample was faced down for the transmission and faced up for the reflection measurements to ensure practical illumination conditions for liquid-state and solid-state experiments. The observed fringes in the measured absorption spectra are due to the Fabry-Perot interferences[7] in the high refractive index GaN layer on the sapphire substrate.[5] All the Au ND array heterostructures show a dipole plasmon resonance mode in the intraband region (1.65 -1.75 eV) which enables us to disentangle plasmon absorption and interband excitation effects.

Electromagnetic Modelling

The absorption spectra of the nanoantennas in air were also calculated using electromagnetic simulations (RF module of COMSOL Multiphysics v5.6, **Figure S1.b**, dashed lines). A standard approach for periodic structures[3,8] was followed, adapted to the specific materials and geometry of our system. Briefly, we employed a 3D unit cell model,[2] consisting of one Au nanoantenna (disk or stripe geometry) on GaN/ Al_2O_3 substrate surrounded with a top layer of air (or water, see below), by setting the unit cell width equal to the array periodicity (200 nm for disks and 230 nm for stripe) and unit cell length equal to 200 nm for disks and 300 nm for stripes, respectively. Perfect magnetic conductor and perfect electric conductor boundary conditions were applied at the sidewalls of the unit cell. Port boundary conditions with excitation “ON” was set at the bottom of the unit cell for the back illumination with a normal incident plane wave (450-850 nm), with electric field polarization perpendicular to the disk diameter and for recording the reflected wave. A second port boundary condition without excitation was used at the top of the unit cell to record

the transmitted wave. Absorbed power was calculated by volume integration of the electromagnetic power loss density over the nanoantenna volume. The wavelength-dependent complex refractive indices for Au, GaN, and Al_2O_3 were taken from Refs. [9], [10], and [11], respectively.

Absorption in Water and Error Calculation

Measured absorptions in air are in good agreement with numerical simulations (**Figure S1.b**). Based on this agreement, we can use the numerical model to calculate the absorption spectra of the samples in an aqueous medium (**Figure S1.c)**, replacing otherwise challenging microscale absorption measurement in liquid. These absorption spectra are subsequently used for the calculation of the internal quantum efficiency (IQE) of the sample. It is to note that the fringes present in the measured absorption spectra in air (**Figure S1.b**), are due to the Fabry-Perot interference[7] within the planar GaN/sapphire substrate structure. Since the photo-SECM experiments were performed under a wide bandwidth illumination of 20 nm, a period of these oscillations is covered. Therefore, using the average (i.e. modeled) absorption values is reasonable for our IQE calculations.

From the comparison between the measured and calculated absorption spectra in air, it is possible to calculate an error on the estimated absorption:

$$\frac{\Delta A}{A} = \frac{A_{exp} - A_{sim}}{A_{exp}} \qquad \text{(S1)}$$

This uncertainty is then accounted for the calculated spectra in water and included in the error analysis for the internal quantum efficiency calculations (**equation S5**).

Supplementary Information 3 – Photochemical Characterization, EQE and IQE Calculations

Photochemical measurements are performed using photo-SECM according to a previously established methodology for plasmonic studies.[2,12] *Below we provide specific details of our set-up and experimental conditions as well as important details concerning the data post-processing steps. Indeed, for transparency and for the reader's convenience we prefer to repeat this information here rather than referring to the associated references.*

Photo-SECM Set-up

We utilized a custom-built photo-SECM set-up, which was integrated by adding a home-built electrochemical reaction cell, a bi-potentiostat (Biologic SP-300), and an ultramicroelectrode (UME) tip connected to a micro/piezo scanner assembly (MMP1/Nano-F450, Mad City Labs) to an inverted optical microscope. The fabrication process of the UME tip is described in our previous work.[2] We used an aqueous electrolyte solution containing 4mM ferricyanide ($Fe(CN)_6^{3-}$) and 0.25M KCl and positioned a 2.9 µm Pt UME tip at a distance of 3 µm from the substrate. We performed the photo-SECM experiments in substrate generation/tip collection (SG/TC) mode, while the sample was illuminated from the bottom with a 30 µm diameter collimated beam. A high power supercontinuum white light laser (NKT Photonics) was utilized for plasmon excitation. The excitation wavelength and power were modulated using a tunable wavelength filter (SuperK VARIA), covering a wide spectral range of 450 to 840 nm with a bandwidth of 20 nm. An optical shutter (SH1, Thorlabs) was employed to block the incident light.

Redox Couple Characterization and Open-Circuit Potential

We verified that the chosen redox couple does not absorb in the studied wavelength range.

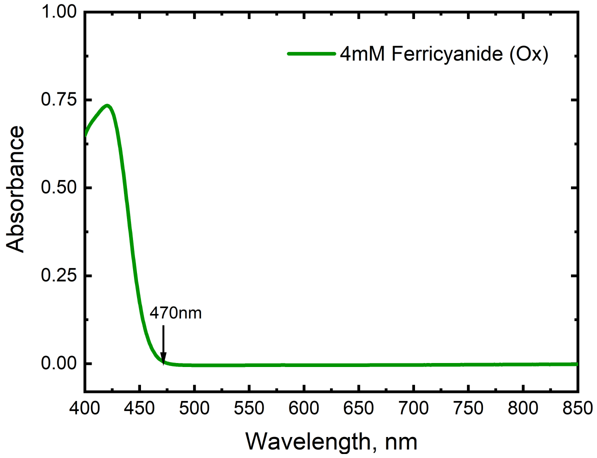

Figure S2. UV-Vis spectrum of 4mM $Fe(CN)_6^{3-}$ in 0.25M KCl aqueous solution. Ferricyanide molecule does not absorb in the wavelength range of 470-850 nm.

We also measured the Fermi level (E_F) of our $Fe(CN)_6^{3-}$/Au/p-GaN system and the LUMO level of the $Fe(CN)_6^{3-}$molecule by open-circuit potential and cyclic voltammetry measurements in the electrolyte media. A value of ≃ 0.31 V vs Ag/AgCl (-4.96 eV vs vacuum), and 0.125 V vs Ag/AgCl (-4.77 eV vs vacuum) was measured for the E_F and LUMO levels, respectively (**Figure S3**).

Therefore, we measure an energy difference between the LUMO and E_F levels of LUMO-E_F = 0.19 eV.

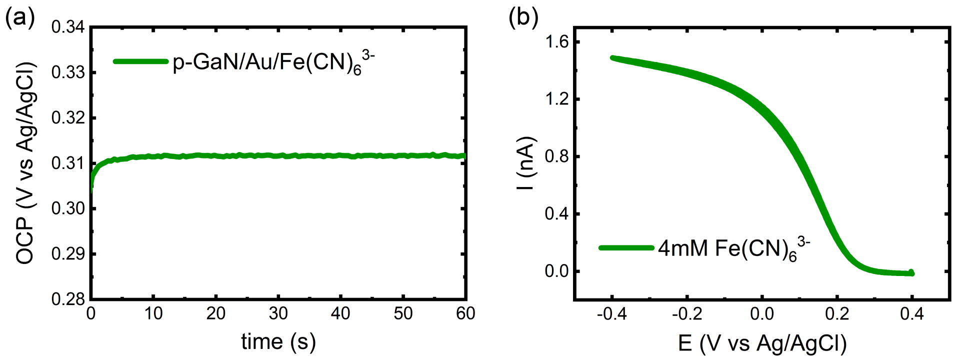

Figure S3. (a) Open-circuit potential (OCP) plot showing the E_{OCP} of Au/p-GaN working electrode in the presence of 4mM $Fe(CN)_6^{3-}$ after 30 min equilibrium time delay. (b) Steady-state cyclic voltammogram of a 2.9 µm UME tip showing the LUMO level of $Fe(CN)_6^{3-}$ that can be estimated from the half-wave potential.

Photo-SECM Data Acquisition and Post-processing

Figure S4.a shows the time-trace of the tip current (i_{Tip}) upon illumination of the 14 nm thick ND array (**Figure S1.a**, SEM image) at a broad excitation wavelength range of 470-832 nm, where the intensity was modulated up to 14 W/cm^2. At this power intensity range, we showed no local heating effect (sharp transients). This is supported by both experimental and theoretical confirmation (see our previous work).[2] The measured tip current relative to the dark current ($i_{Tip}/i_{Tip,dark}$) under different excitation intensities at each wavelength is then calculated (**Figure S4.b**). We observe a linear increase in the magnitude of the ($i_{Tip}/i_{Tip,dark}$) by increasing the excitation intensity at each excitation wavelength. This increasing trend indicates that the local concentration of $Fe(CN)_6^{4-}$ at the tip-substrate gap increases due to an electron-driven reduction reaction at the plasmonic substrate whose kinetics gets enhanced by the illumination intensity. A 2D diffusion COMSOL model was also implemented to simulate the tip current (i_{Tip}) response in photo-SECM experiments for a photo-reduction reaction, obtaining the calibration curve shown in (**Figure S4.c**). Simulation details are provided in our previous work.[2] For each excitation wavelength we extracted the substrate photocurrent ($i_{sub,photo}$) as a function of power using the calibration curve and the measured $i_{Tip}/i_{Tip,dark}$ under different illumination intensities (**Figure S4.d)**.

The external quantum efficiency (EQE) of the photoelectrochemical reaction (**eq. S2**) is determined from the slope of the linear fit to the $i_{sub,photo}$ vs illumination power curves for each excitation wavelength (**Figure S4.d**) using **equation S3**.

$$Fe(CN)_6^{3-}\ (Ox) + e^- \rightarrow\ Fe(CN)_6^{4-}\ (Red) \tag{S2}$$

$$EQE = \frac{i_{sub,photo}/e}{P/hv} \tag{S3}$$

where $i_{sub,photo}/P$ is the slope of the substrate photocurrent vs power plot, e is the charge of electron, and hv is the photon energy.

The internal quantum efficiency (IQE) and ΔIQE are determined using **equations S4** and **S5**, respectively.

$$IQE = \frac{EQE}{A} \tag{S4}$$

$$\Delta IQE = IQE\sqrt{(\frac{\Delta EQE}{EQE})^2 + (\frac{\Delta A}{A})^2} \tag{S5}$$

where A is absorption and Δ corresponds to the error in a quantity.

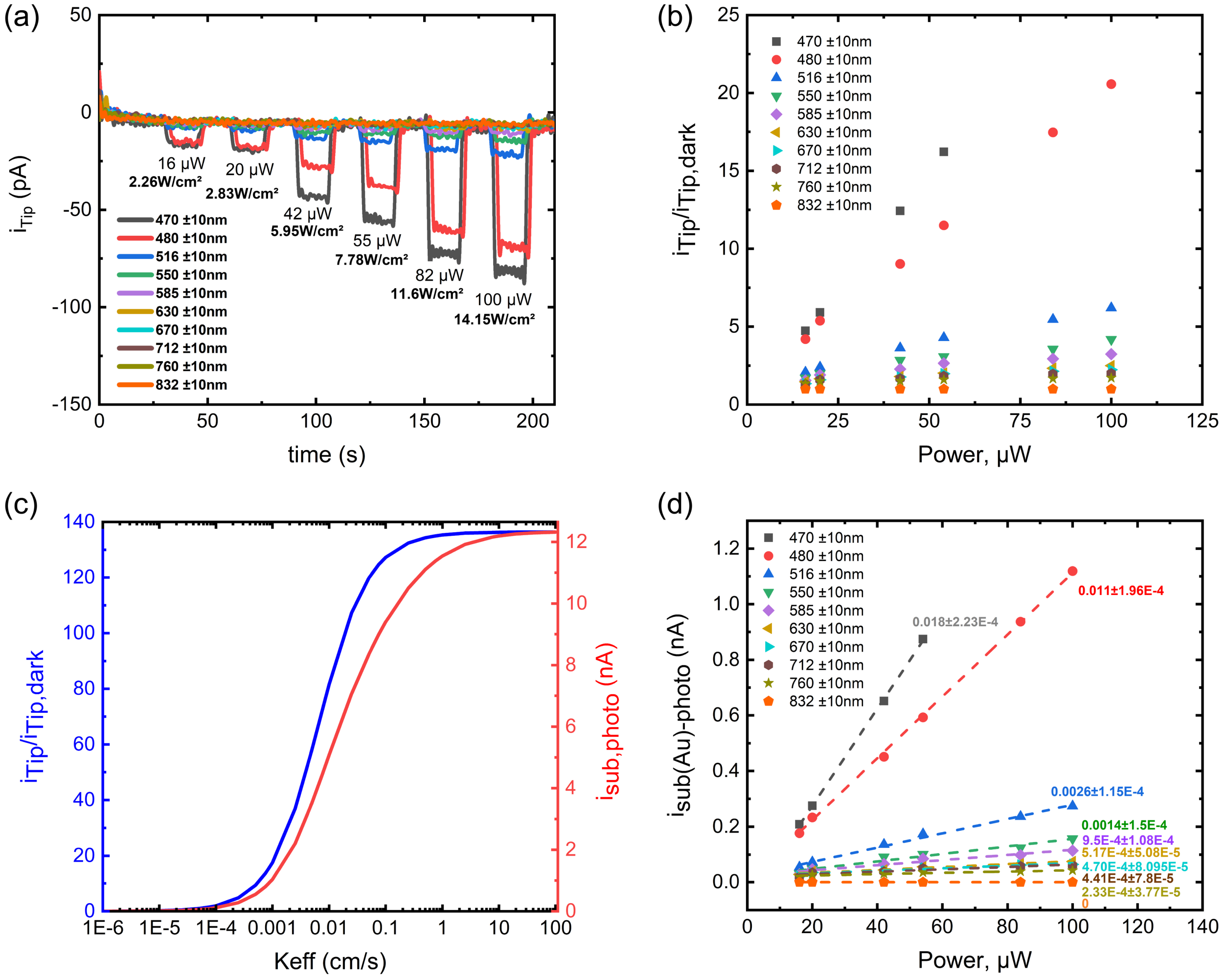

Figure S4. (a) Time trace of tip current (i_{Tip}) obtained from a 1.45 µm radius Pt UME in an SG/TC SECM mode upon illumination of the 14 nm thick Au NDs array with the excitation wavelength of 470-832±10 nm up to 100 µW (power intensity of 14.15 W/cm^2). The tip-to-substrate distance was 2.5 µm. (b) Measured $i_{Tip}/i_{Tip,dark}$ response as a function of power for a broad excitation wavelength range of 470-832 nm. (c) Simulated calibration curves correlating $i_{Tip}/i_{Tip,dark}$, $i_{sub,photo}$, and k_{eff} obtained by the COMSOL diffusion model reported in our previous work.[2] (d) Extracted substrate photocurrent ($i_{sub,photo}$) vs power, using the calibration curves in (c) and measured data of $i_{Tip}/i_{Tip,dark}$ in (b) for each excitation wavelength. The slope of the linear fit (dashed lines) to these data is mentioned for each excitation wavelength.

As a control experiment, we performed the same SECM measurement on a bare p-GaN substrate in the absence of the Au NDs. An extremely low photo-induced (i_{Tip}) enhancement was observed at short wavelengths (470 nm-480 nm) by increasing the excitation intensity (**Figure S5**). This was therefore subtracted from the Au/p-GaN photocurrent.

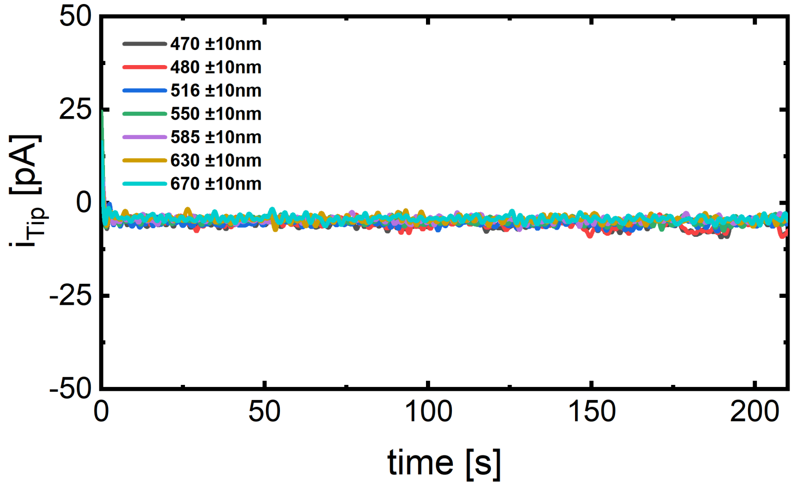

Figure S5. Measured i_{Tip} under illumination of p-GaN substrate with excitation wavelengths of 470-670±10 nm.

Supplementary Information 4 – Au/p-GaN Photodiode Properties and Characterization

For verification purposes aimed at understanding the behavior of the solid/solid interface, we fabricated an Au/p-GaN photodiode. The nanofabrication process was the same as for the Au ND structure. Two modification were necessary:

- Use of stripes to perform current collection
- Use of FIB to etch the strips.

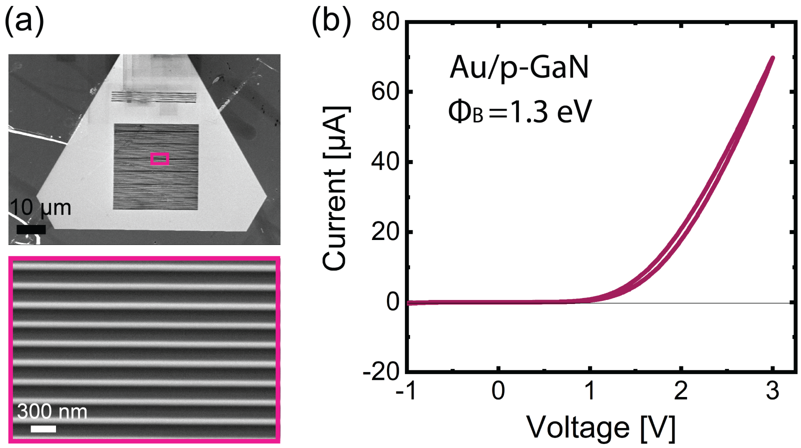

Figure S6. (a) SEM image shows a 30x30 µm^2 stripe array from a 15 nm-thick Au MF together with the higher magnification SEM image of the fabricated Au stripe array. The average stripe width and thickness are 75 and 15 nm, respectively. The array periodicity is 230 nm. (b) Measured I-V plot of the fabricated heterostructure showing a metal-semiconductor Schottky diode behavior across the Au/p-GaN interface. A Schottky barrier height of Φ_B =1.3 eV was estimated after fitting these data.

SEM image in **Figure S6.a** shows a fabricated 30x30 µm^2 stripe array together with the magnified view of the fabricated stripe structure with a width of 75 nm and periodicity of 230 nm.

Absorption measurements were performed according to the method described in supplementary information 2, and adding a polarizer to the optical path to provide a polarized beam to the Au stripes. **Figure S8** shows measured absorption and EQE spectra of the fabricated Au stripe/p-GaN heterostructures.

For the solid-state photocurrent measurements we used piezoelectric microprobes (Imina Technologies, miBots™) to electrically connect the sample and record the short-circuit current. We added a polarizer (WP25M-VIS, Thorlabs) to the optical path to provide a polarized beam perpendicular to the Au stripes.

The current-voltage (*I-V*) and time-trace of the photocurrent (*I-t*) curves were recorded through a Keithley 2450 SourceMeter. From the I-V response shown in **Figure S6.b**, we estimated a Schottky barrier of ≃ 1.3 eV across the Au/p-GaN heterojunction.

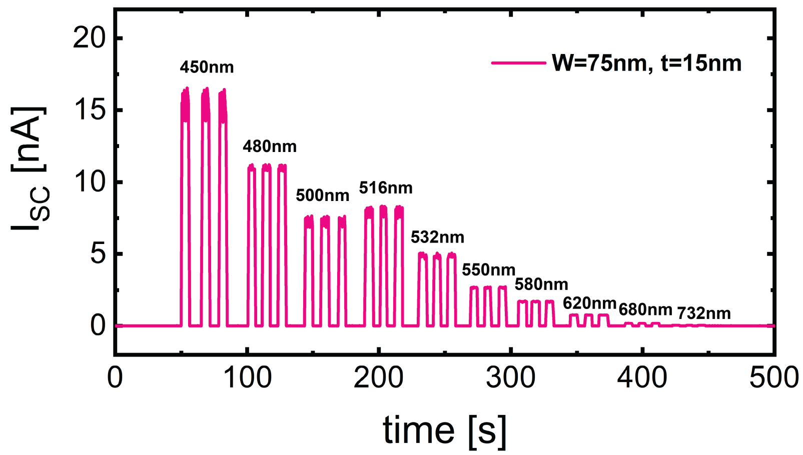

Figure S7. Time-trace of the short-circuit current (I_{SC}) for the fabricated stripe array in **Figure S4.a** upon illumination with the excitation wavelengths of 450-800±10 nm.

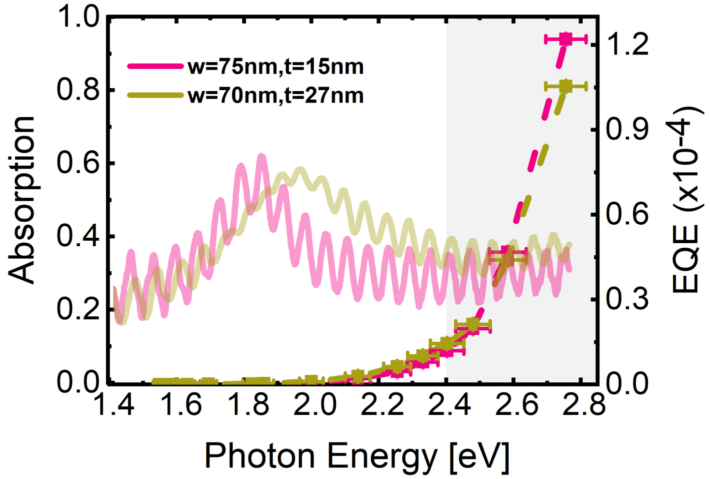

Figure S8. Measured absorption and EQE spectra of the fabricated Au stripe/p-GaN heterostructures having thicknesses of 15 and 27 nm. The gray shaded areas depict the purely interband region and the dashed lines are a guide to the eye.

Supplementary Information 5 – Hot carrier transport and injection, Detailed IQE analysis

The carrier fluxes incident on the surfaces of the structure before and after scattering events were predicted using the Non-Equilibrium Scattering in Energy and Space (NESSE) simulation framework.[2,13] Briefly, this technique uses *ab initio* simulations of the optical excitation due to direct and phonon-assisted transitions to predict the initial carrier distribution, and then evolves spatially-resolved carrier energy distribution using the Boltzmann transport equation with a collision integral parameterized to first-principles electron-phonon and electron-electron scattering. The NESSE formulation predicts the carrier flux incident on the surfaces of the structure before scattering, after scattering once, twice, etc., allowing for the separation of contributions due to scattered and unscattered carriers. Further details of the algorithm and the underlying first principles calculations used to parameterize carrier generation and transport parameters are provided in Ref. [7]

Combining the experimental internal quantum efficiency data and the calculated hot electron fluxes, we previously developed an approach to extract the electron injection probability from the Au nanoantenna, $P_{inj}(E)$. By assuming that $P_{inj}(E)$ is constant for all samples (as the same surfaces are exposed) and is only a function of the electron energy when an electron reaches a surface (i.e. not a function of incident photon energy), we can state that for the NDs:

$$IQE(\hbar\omega) = \int_{E_F}^{\infty} (F_{N,top}(E,\hbar\omega)P_{inj,top}(E) + F_{N,side}(E,\hbar\omega)P_{inj,side}(E))dE \qquad (S5)$$

Here $\hbar\omega$ is the photon energy, E is the energy of the hot electrons with respect to E_F, N is the number of the scattering events we include in modeling and top/side subscripts refer to the top/side surfaces of the structure. As we know $F_N(E, \hbar\omega)$, we can theoretically calculate IQEs based on an assumed $P_{inj}(E)$. We developed a stochastic fitting procedure which varied $P_{inj}(E)$ to minimize the difference between experimental and calculated IQEs. The details of this approach are provided in our previous work.[2] By performing this fitting procedure hundreds of times we were able to calculate the average and range of possible $P_{inj}(E)$ values. The resulted $P_{inj}(E)$ from this fitting approach for the top and side surfaces is shown in **Figure 4.c** and **Figure S9**.

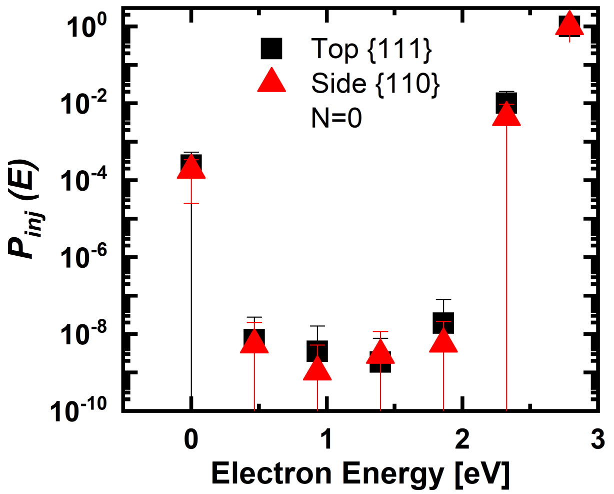

Figure S9. Injection probability (P_{inj} *(E)*) for hot electrons ballistically collected (no scattering, N=0) from the top {111} and side {110} facets of the Au ND heterostructures.

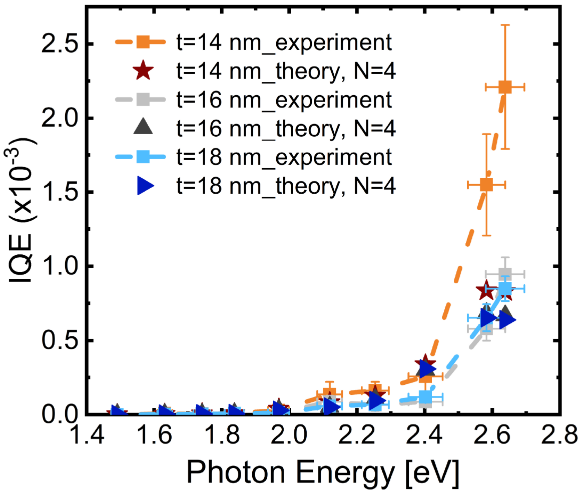

Figure S10. Calculated IQE spectra based on energy-resolved hot electron fluxes and estimated P_{inj} *(E)* in **Figure 4.c**, together with the experimentally determined IQEs for Au ND heterostructures. Dashed lines are a guide to the eye.

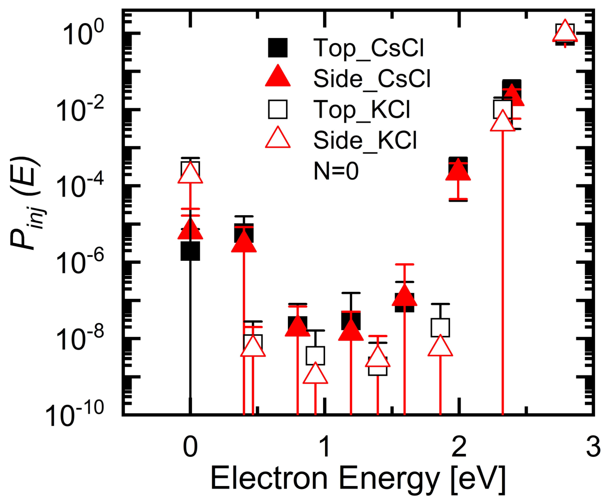

Figure S11. Calculated injection probabilities for the measurements performed in the CsCl electrolyte (filled symbols) together with the measurements in the KCl electrolyte (empty symbols). The hot electrons collected ballistically (N=0) from the top {111} and side {110} facets of the Au ND heterostructures.

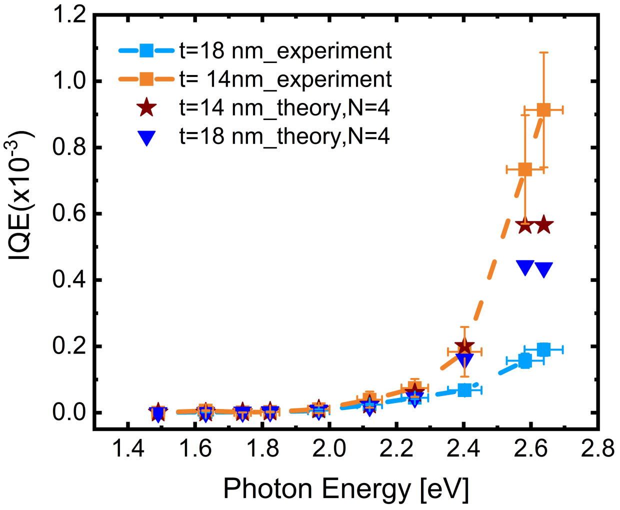

Figure S12. Calculated IQE spectra based on energy-resolved hot electron fluxes and estimated Pinj (E) in **Figure 4.d**, together with the experimentally determined IQEs for Au ND heterostructures in CsCl electrolyte. Dashed lines are a guide to the eye.

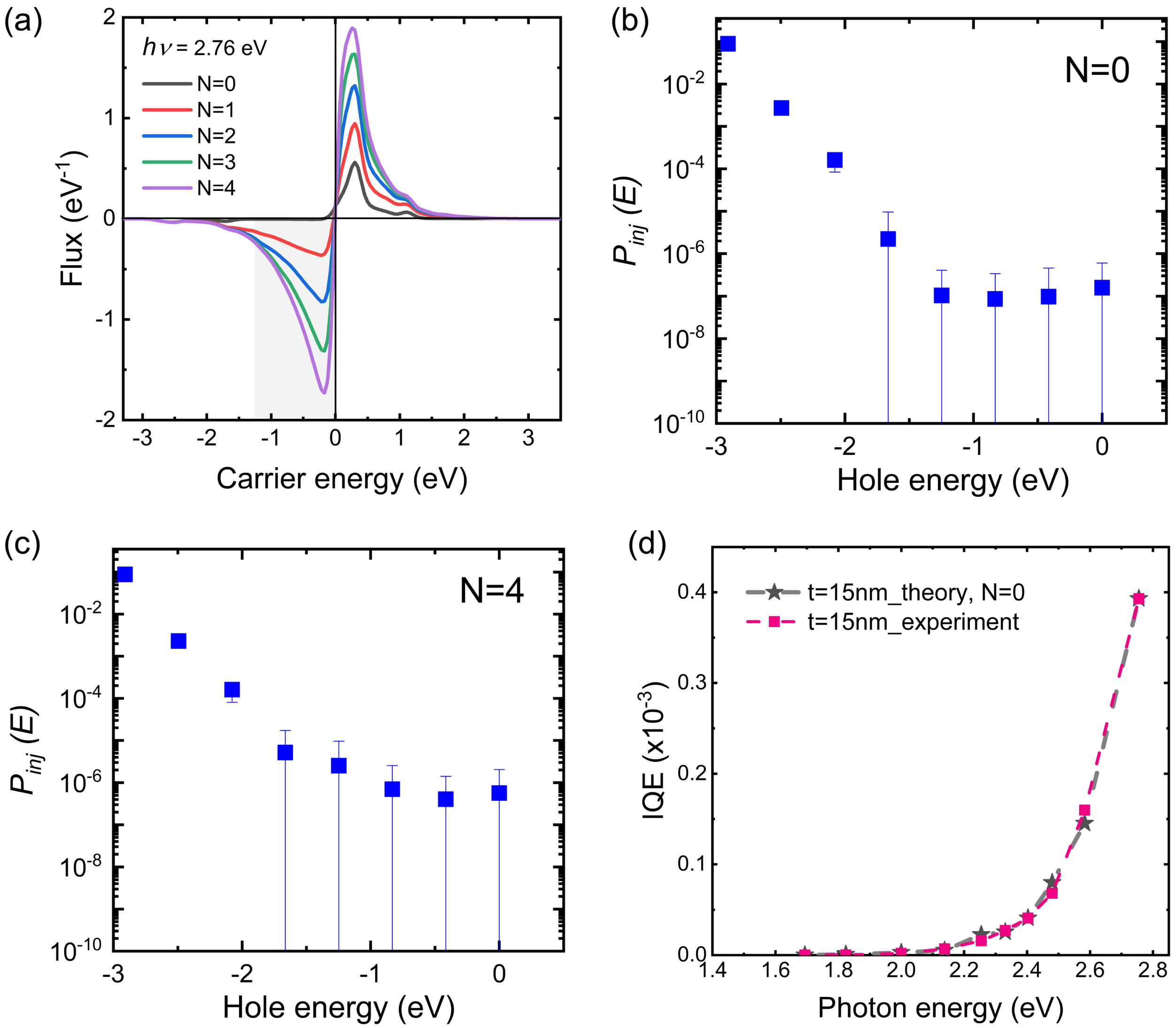

Figure S13. (a) Calculated energy-resolved carrier fluxes reaching the bottom surface of 15 nm thick Au stripe directly (N=0) or upon scattering (N=1-4) under illumination at 450 nm (2.76 eV). The carrier energies are referenced to the Au Fermi level located at 0 eV. Negative values of hot-carrier energy correspond to hot holes (left side) and positive values correspond to hot electrons (right side). The gray shaded area shows the position of the Schottky barrier (1.3 eV). (b) Estimated injection probability (P_{inj} *(E)*) for hot holes collected from the bottom interface of the Au stripe (b) directly (N=0), or (c) via scattering (N=4). (d) Calculated IQE spectrum based on energy-resolved hot hole fluxes and estimated P_{inj} *(E)* in (b), together with the experimentally determined IQE for Au/p-GaN photodetector. Dashed lines are a guide to the eye.

To compare the experimentally IQE obtained from our solid-state photocurrent measurements with our model, the same transport model was employed considering the Schottky barrier height of 1.3 eV across

the Au/p-GaN interface and assuming tangential momentum conservation for estimating the Pinj(E). **Figure S13.a** shows the calculated carrier fluxes (negative energy axis for holes) that reach the bottom interface directly or upon scattering (up to 4 events scattering) under illumination at 450 nm (2.76 eV) for the 15 nm thick stripe heterostructure. The obtained Pinj (E) for the hot holes that reach the bottom interface directly (N=0) or via scattering (N=4) is plotted in **Figure S13.b,c**, exhibiting almost the same exponentially growing probability and the maximum Pinj (E) for the high-energy d-band holes. This indicates that these high-energy holes that have enough energy to overcome the Schottky barrier undergo no scattering before being collected at the Au/p-GaN interface. Importantly, the calculated IQE for nonscattered carriers (N=0) in **Figure S13.d** shows an excellent agreement with the experimental IQE, further confirming the ballistic collection of the hot holes in our system. In fact, the occurrence of the largest carrier generation at this interface and the suitable design of our nanoantenna thickness, favor this ballistic collection.

References

(1) Kiani, F.; Tagliabue, G. High Aspect Ratio Au Microflakes via Gap-Assisted Synthesis. *Chem. Mater.* **2022**, *34* (3), 1278–1288. https://doi.org/10.1021/acs.chemmater.1c03908.

(2) Kiani, F.; Bowman, A. R.; Sabzehparvar, M.; Karaman, C. O.; Sundararaman, R.; Tagliabue, G. Transport and Interfacial Injection of D-Band Hot Holes Control Plasmonic Chemistry. *ACS Energy Lett.* **2023**, 4242–4250. https://doi.org/10.1021/acsenergylett.3c01505.

(3) Tagliabue, G.; Jermyn, A. S.; Sundararaman, R.; Welch, A. J.; DuChene, J. S.; Pala, R.; Davoyan, A. R.; Narang, P.; Atwater, H. A. Quantifying the Role of Surface Plasmon Excitation and Hot Carrier Transport in Plasmonic Devices. *Nat. Commun.* **2018**, *9* (1), 1–8. https://doi.org/10.1038/s41467-018-05968-x.

(4) Jiao, L.; Fan, B.; Xian, X.; Wu, Z.; Zhang, J.; Liu, Z. Creation of Nanostructures with Poly(Methyl Methacrylate)-Mediated Nanotransfer Printing. *J. Am. Chem. Soc.* **2008**, *130* (38), 12612–12613. https://doi.org/10.1021/ja805070b.

(5) Tagliabue, G.; DuChene, J. S.; Habib, A.; Sundararaman, R.; Atwater, H. A. Hot-Hole versus Hot-Electron Transport at Cu/GaN Heterojunction Interfaces. *ACS Nano* **2020**, *14* (5), 5788–5797. https://doi.org/10.1021/acsnano.0c00713.

(6) Bowman, A. R.; Ma, J.; Kiani, F.; García Martínez, G.; Tagliabue, G. Best Practices in Measuring Absorption at the Macro- and Microscale. *APL Photonics* **2024**, *9* (6), 061101. https://doi.org/10.1063/5.0210830.

(7) Pérot, A.; Fabry, C. On the Application of Interference Phenomena to the Solution of Various Problems of Spectroscopy and Metrology. *Astrophys. J. Vol 9 P 87* **1899**, *9*, 87.

(8) Tagliabue, G.; Eghlidi, H.; Poulikakos, D. Facile Multifunctional Plasmonic Sunlight Harvesting with Tapered Triangle Nanopatterning of Thin Films. *Nanoscale* **2013**, *5* (20), 9957–9962. https://doi.org/10.1039/C3NR03273F.

(9) Olmon, R. L.; Slovick, B.; Johnson, T. W.; Shelton, D.; Oh, S.-H.; Boreman, G. D.; Raschke, M. B. Optical Dielectric Function of Gold. *Phys. Rev. B* **2012**, *86* (23), 235147. https://doi.org/10.1103/PhysRevB.86.235147.
(10) Kawashima, T.; Yoshikawa, H.; Adachi, S.; Fuke, S.; Ohtsuka, K. Optical Properties of Hexagonal GaN. *J. Appl. Phys.* **1997**, *82* (7), 3528–3535. https://doi.org/10.1063/1.365671.
(11) Zhukovsky, S. V.; Andryieuski, A.; Takayama, O.; Shkondin, E.; Malureanu, R.; Jensen, F.; Lavrinenko, A. V. Experimental Demonstration of Effective Medium Approximation Breakdown in Deeply Subwavelength All-Dielectric Multilayers. *Phys. Rev. Lett.* **2015**, *115* (17), 177402. https://doi.org/10.1103/PhysRevLett.115.177402.
(12) Yu, Y.; Wijesekara, K. D.; Xi, X.; Willets, K. A. Quantifying Wavelength-Dependent Plasmonic Hot Carrier Energy Distributions at Metal/Semiconductor Interfaces. *ACS Nano* **2019**, *13* (3), 3629–3637. https://doi.org/10.1021/acsnano.9b00219.
(13) Jermyn, A. S.; Tagliabue, G.; Atwater, H. A.; Goddard, W. A.; Narang, P.; Sundararaman, R. Transport of Hot Carriers in Plasmonic Nanostructures. *Phys. Rev. Mater.* **2019**, *3* (7), 075201. https://doi.org/10.1103/PhysRevMaterials.3.075201.
